# When is a postmortem examination carried out? A retrospective analysis of all Swedish deaths 1999–2018

**DOI:** 10.1007/s00428-022-03462-w

**Published:** 2022-11-29

**Authors:** Fredrik Tamsen, Irina Alafuzoff

**Affiliations:** 1 Department of Immunology, Genetics and Pathology, Uppsala University, Uppsala, Sweden; 2 Department of Pathology, Uppsala University Hospital, Uppsala, Sweden

**Keywords:** Autopsy, Cause of death, Clinical pathology, Forensic pathology, Dementia, Gender, Equality

## Abstract

The objective of this study was to assess who is merited an autopsy in Sweden. Data from the Swedish cause of death (COD) registry over a period of 20 years was retrieved and analysed. A multinominal logistic regression analysis was performed to identify the variables that were most strongly associated with the performance of a clinical or forensic autopsy (CA/FA).

A definite COD, i.e. a COD based on autopsy findings, was registered in 12.6% of all deceased during the investigated period. In the remaining cases, the COD was presumed by the clinicians. Being male, born in the Nordic region, dying in a private residence, and unnatural death were most strongly associated with the performance of CA/FA. In contrast, being female, dying from dementia, dying at a nursing home, being born outside of Europe, or living in a small city or rural area seldom led to the performance of CA/FA.

The above is certainly surprising as an autopsy provides an opportunity to investigate the cause of death, validate clinical diagnoses, detect unexpected aberrations, audit health care, and provide feedback to clinicians to facilitate their continuing education.

## Introduction

The objective of a postmortem (PM) examination is primarily to investigate pathological changes and traumatic injuries in order to assess the cause of death (COD). An autopsy report can clarify whether the antemortem (AM) diagnoses were correct and whether AM treatments were relevant. Some disease processes are impossible to assess in vivo, and the clinical diagnosis is based on AM-observed symptoms and signs, for example, as with relatively common cognitive decline (CD). A Swedish study found significant discrepancies in the assigned cause of cognitive decline when comparing AM and PM diagnoses [1]. The clinical diagnosis was fully or partly in accordance with the neuropathological findings in only 63% of the cases. Noteworthy, since 2009, new tissue alterations in the brain causative regarding CD have been identified. Many of these tissue alterations lack diagnostic biomarkers to be used AM, and thus, the AM and PM agreement rate today is probably even lower [2–4].

Another diagnosis that might be missed AM is pulmonary embolism [5]. The symptoms may be misinterpreted as primarily cardiogenic, and embolism can be missed despite using computed tomography (CT). At autopsy however, pulmonary embolism can easily be verified, even by naked eye examination.

Since 2020, the SARS-CoV-2 pandemic has reminded the medical community that PM examinations have a significant role, even in the modern era. It is easy to assess that a deceased is COVID-19-positive, whereas the actual tissue damage being the COD can only be assessed PM [6, 7].

Despite all the above, the number of PM examinations has steadily declined in Western Europe during the last decades [8].

It has been reported repeatedly that PM examinations often reveal major diseases that are unknown AM [9–14]. In some cases, a correct AM diagnosis might have changed the management of care and could have prolonged survival or cured the patient. Such discrepancies have also been seen in patients under close surveillance. In a study that compared the AM and PM diagnosis in a cohort of 126 deceased subjects at an intensive care unit, a new relevant diagnosis that might have changed the therapy was noted PM in 9.5% [15].

Autopsies are also valuable in preterm infant deaths. In a Swedish centre, during a period of 16 years, 278 infants died, of which 203 (73%) were autopsied; in 23.7%, the AM assigned cause of death was completely revised following the PM examination [14]. The authors conclude that autopsies contribute to the development of neonatal care and possibly improved survival in preterm infants.

Last and certainly not the least, a PM examination provides answers to relatives as to why their loved ones died, which can be an important part of the grieving process [16, 17].

In summary, despite advances in diagnostics, the PM examination is still important to reach definite diagnoses.

The aim of this study was to review autopsy practice in Sweden by exploring data from the COD registry. Different characteristics of the deceased and the circumstances of death were analysed with respect to how they were associated with the odds that a PM examination was performed.

## Material and methods

The study was a retrospective explorative analysis. Data from the Swedish national COD registry was extracted anonymously on an individual level for a 20-year period: 1999–2018. The data was originally reported to the COD registry by the clinicians or by the forensic pathologists when an FA had been performed. The variables included in the analysis are listed in Table 1.

**Table 1:** Variables that were analysed from the Swedish cause of death registry

Variable	Possible values	Comment
Year of death	1999–2018	
Age at death	Integer from 0 and up	Divided into age groups for some analyses
Gender	Male; female; unknown	
Postmortem examination	Clinical autopsy; forensic autopsy; only external forensic examination; no autopsy	In the analysis, ‘only external forensic examination’ was regarded as ‘no autopsy’
Underlying cause of death	ICD-10	Only some diagnoses were considered in the analyses
Operated within four weeks prior to death	Yes; no; information missing; not filled in	The variable was not reported in 12% of the deceased
Place of death	Hospital; nursery home; private residence; other/unknown; not filled in	Available from 2003 with much missing data 2003–2006 and around 10% missing data from 2007 and onwards. The variable was not reported in 34% of the deceased
Municipality of residency	Municipality code	Grouped into three classes^1^: A: large cities including adjacent municipalities B: medium cities including adjacent municipalities C: small cities including adjacent municipalities and rural areas
Country of birth	Sweden, Nordic countries (excluding Sweden), Europe (excluding Nordic countries), Africa, Asia, North America, South America, Oceania, and others (few)	In the analyses, the values were grouped as Sweden, Nordic region excluding Sweden, Europe excluding Nordic region, and outside Europe. There were few missing data

^1^https://skr.se/skr/tjanster/kommunerochregioner/faktakommunerochregioner/kommungruppsindelning.2051.html

In Sweden, a CA can be requested by a clinician when death is presumed to be natural, and the autopsy is carried out by a clinical pathologist. An FA is carried out by a forensic pathologist upon request from the police authority when death can be suspected to be unnatural. An FA, instead of a CA, should also be performed when there is a suspicion of an iatrogenic lethal event in the health care organisation. Noteworthy, a CA usually requires consent from the relatives of the deceased, as opposed to an FA. Specific investigations such as a neuropathological examination are performed when the clinician asks for it or the pathologist considers it to be relevant regarding the COD.

The relationship between different variables and PM examination was analysed with multinomial logistic regression, with PM examination as the dependent variable and the other variables as independent. Odds ratio (OR) was used to assess the impact of a variable on whether a CA or FA had been performed. Since the study includes all deaths in Sweden from the years in question, any difference found is real in absolute terms. However, to assess whether such differences between groups may be due to chance, the data was viewed as a virtual sample. A *p* value of less than 0.05 was considered statistically significant. The statistical analyses were performed by the statistical bureau Statisticon.

## Results

A total of 1,837,042 subjects (48.6% men and 51.4% women) were registered in the Swedish COD registry during the 20-year-long period (1999–2018). Out of all deaths during this period, PM examinations were carried out in 12.6% of the subjects: 6.9% were referred for a CA and 5.7% for an FA.

The rate of CA has steadily declined, being halved for both sexes during the study period. For men, the rate has gone from around 12% in 1999 to just below 6% in 2018; for women, the drop has been from over 8% to under 4%. The frequency of FA has been relatively stable, with 8–9% for men and around 3% for women.

Table 2 shows the results from the multinomial logistic regression, including all the analysed variables. All variables are statistically significantly associated with whether a CA or an FA has been performed, respectively.

**Table 2:** Odds ratio for the performance of an autopsy—multinomial logistic regression with all analysed variables

		Clinical autopsy		Forensic autopsy	
Variable	Value	Odds ratio	95% CI	Odds ratio	95% CI
Age		0.96	0.96–0.96	0.94	0.94–0.94
Year		0.96	0.96–0.96	1.00	1.00–1.01
Place of death	Unknown	0.95	0.93–0.96	5.66	5.47–5.86
	Private residence	1.90	1.87–1.94	25.88	25.05–26.73
	Nursing home	0.23	0.23–0.24	0.60	0.56–0.64
Country of birth	Europe	0.73	0.71–0.76	0.82	0.79–0.86
	Outside Europe	0.42	0.40–0.45	0.51	0.48–0.54
	Nordic Region	1.14	1.12–1.17	1.71	1.66–1.77
Gender	Male	1.32	1.31–1.34	2.26	2.22–2.31
Surgery	No	0.75	0.74–0.77	8.39	7.86–8.95
	Unknown	0.91	0.88–0.83	19.92	18.64–21.28
Municipality	B	1.03	1.01–1.04	0.76	0.75–0.78
	C	0.94	0.92–0.94	0.59	0.57–0.60
COD	External	0.65	0.62–0.68	58.66	57.15–60.22
	Dementia	0.24	0.22–0.26	0.08	0.06–0.11

Dependent variable with reference category within parentheses: postmortem examination (no autopsy). Independent variables with reference categories within parentheses: place of death (hospital); country of birth (Sweden); gender (female); surgery, operated within four weeks prior to death (yes); municipality of residency (A); COD, underlying cause of death (other). All odds ratios differed statistically significantly from the reference categories with *p* value < 0.001. Age and year are included as continuous variables

The factors that have the strongest associations with a CA being performed are death in a private residence, being male, or born in the Nordic region. By contrast, dying from dementia, at a nursing home, or being born outside of Europe have the strongest associations with a CA not being performed. The performance of an FA is most strongly associated with unnatural death, death in a private residence, or when it is unknown whether surgery has been performed recently. Dementia as the underlying COD, birthplace outside of Europe, or living in a small city or rural area (type C municipality) are most strongly associated with an FA not being performed.

**Fig. 1:**
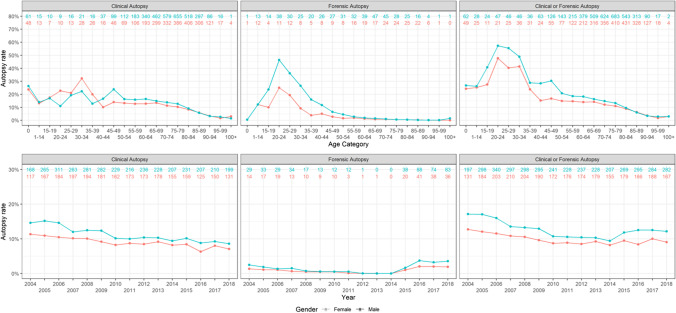
Autopsy frequency in the subgroup of newly operated that died in hospitals. Results are shown for men and women separately, both divided into age groups (< 40 years, 40–79 years, and ≥ 80 years) and for all ages over the years 1999–2018. Numbers at the top of the graphs refer to the absolute numbers of subjects

Figure 1 reflects a subgroup of those who died in hospitals after a recent operation. When looking at CA/FA autopsies together, men had significantly higher odds of undergoing an autopsy when compared to females. To analyse the influence of age, the population was divided into three larger age groups: < 40 years, 40–79 years, and ≥ 80 years. Men were more often subject to a CA in the middle and highest age groups, with OR 1.27 (95% CI, 1.19–1.36) and 1.12 (95% CI 1.01–1.23), respectively. In the lowest and middle age groups, FA was more likely for men compared to women, with odds ratio (OR) 2.49 (95% confidence interval, CI 1.80–3.50) and 1.59 (95% CI 1.29–1.98), respectively.

**Fig. 2:**
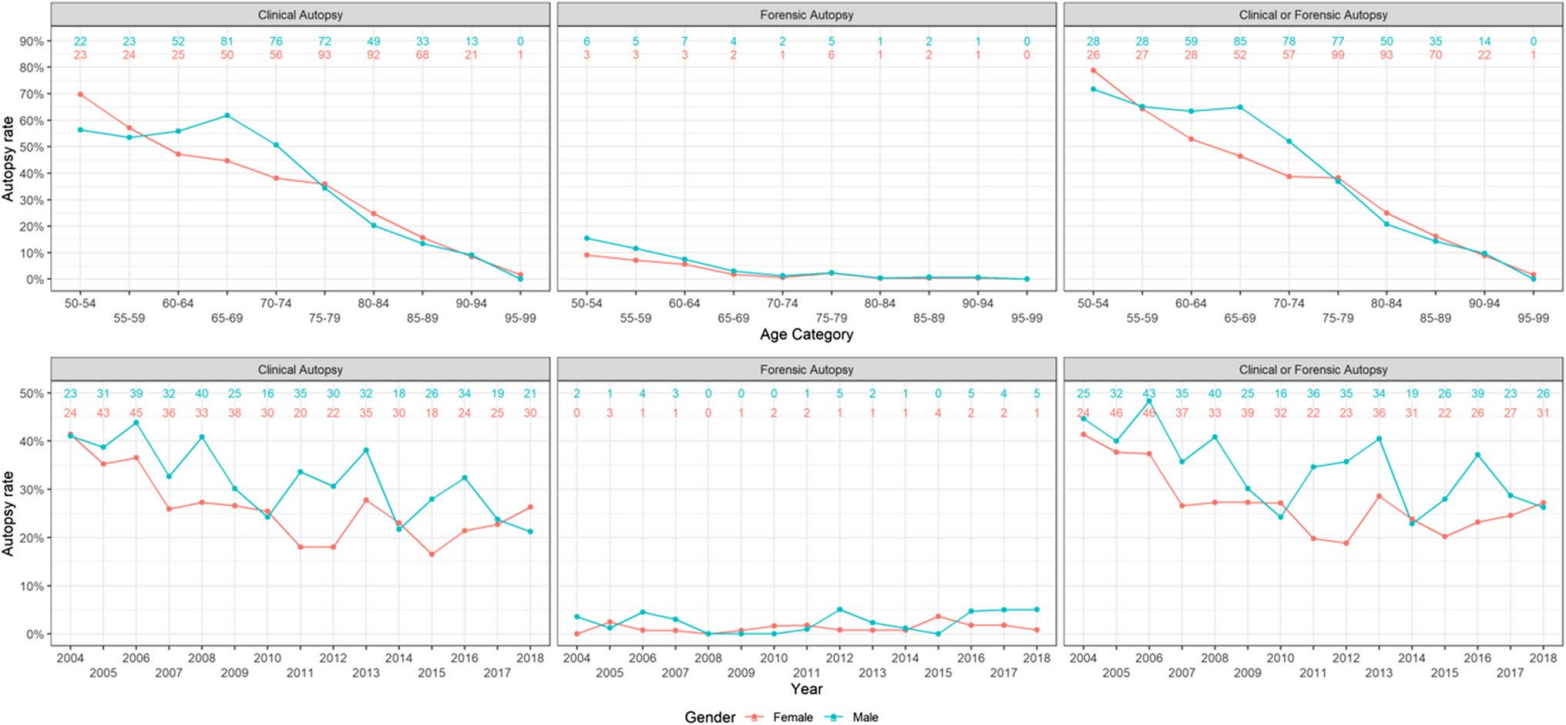
Autopsy frequency in the subgroup that died from pulmonary embolism in hospitals. Results are shown for men and women separately, both divided into age groups and as development over the years 1999–2018. Numbers at the top of the graphs refer to the absolute numbers of subjects

The subgroup of people who died from pulmonary embolism in hospitals is shown in Fig. 2. There is a clear decline of CA rate with age. There were no statistically significant differences between men and women for CA/FA autopsies.

When the underlying COD was cognitive decline and dementia, the odds that an autopsy was performed were much lower when compared to other causes of death. The group with Alzheimer’s disease, vascular dementia, Parkinson’s disease, or unspecified dementia as underlying COD was compared with all other deaths. The comparison was made separately for men and women and adjusted for age and year of death. The OR was 0.09 (95% CI 0.08–0.11) for men and 0.12 (95% CI 0.11–0.14) for women. When looking at the four dementia diagnoses separately, the ORs were similarly low with statistically significant differences between those with and without the respective diagnosis as the underlying COD.

## Discussion

The general decline in performed PM examinations is well known and has been discussed for decades [18–22]. During the currently studied 20-year period, only 12.6% of all deceased received a definite COD, i.e. a COD based on autopsy findings. The present study aimed to further identify parameters that might influence the eventuality of PM examinations in a developed country with community-based health care system such as Sweden. To our knowledge, this type of analysis has only been performed in one previous study, albeit with partly different variables and not including the important factor of the underlying COD [23].

Men were in general more likely to be PM investigated with an autopsy. A hypothetical explanation to the gender difference in general could be the extent to which men and women have contact with the health care system. If women seek help for their medical problems more frequently than men, their state of health will be better known when they die; thus, the doctor may decide that an autopsy is not necessary to assign the COD. Another possible explanation is that men for some unwarranted reason are more thoroughly investigated when they die. However, the present study does not have any data to support or refute such speculations.

This gender difference was also seen in the subgroup of newly operated. A higher rate of FA for men compared to women might be explained by that men more often suffer from traumatic deaths, which may be preceded by acute trauma surgery. The higher rate of CA for men is harder to explain. Both men and women referred for surgery on clinical grounds have been in recent contact with the health care; thus, their health states should be equally well known. The observed difference of CA might suggest that women are less thoroughly investigated when they die shortly after an operation.

Dementia as the underlying COD had the strongest association with not being investigated with an autopsy. We can thus conclude that in the vast majority of subjects that die from dementia, the diagnosis is primarily based on AM clinical assessment. As previously mentioned, a significant discrepancy has been found between AM and PM assigned diagnoses of neurodegenerative diseases [1]. This is not surprising, as the clinical assessment of a subject with cognitive impairment lacks reliable biological markers to be used AM. In the diagnostics of Alzheimer’s disease, assessment of cerebrospinal fluid for hallmark proteins such as hyperphosphorylated tau and β-amyloid (Aβ) might be carried out. Furthermore, in some centres, the positron emission tomography visualising Aβ in the brain is performed. However, most culprit proteins causing neurodegeneration are not detectable in the blood, CSF, or by means of imaging. A consequence is thus that clinical treatment trials that are oriented towards culprit proteins will rely on AM clinical diagnoses and thus will eventually lead to unreliable results, or no results at all. Accordingly, autopsies with neuropathological examinations are of interest to assign a definite diagnosis to validate the clinically presumed diagnosis. The definite diagnosis is also of interest to relatives to identify eventual hereditary disease. Moreover, the definite diagnosis is certainly of interest for research and the development of new treatments. A hypothetical explanation for the low number of autopsies performed on subjects with clinically presumed brain disease might be the current lack of active treatments of neurodegenerative diseases.

The finding that small cities and rural areas (municipality C) have a lower odds ratio for CA might be explained by that they are served by smaller hospitals and not university hospitals, where the autopsy practice is probably more easily available.

Being born outside of Europe was one of the factors that had the strongest associations with a CA/FA not being performed. The outcome is probably associated with cultural aspects. In Sweden, the CA is requested by a clinician and in most cases performed only after the relatives have given their consent. When relatives deny the autopsy due to religious convictions, it is most often respected. The number of immigrants has increased significantly in Sweden during the last two decades. In 2018, 19% of the population was born in another country, and 11% was born outside of Europe [24]. Cultural objections to autopsies may therefore become an increasingly important factor when analysing the autopsy frequency in the future.

Most deaths in Sweden, as in the rest of the EU, are not PM investigated. This means that the COD statistics is mostly based on diagnoses presumed by clinicians and a substantial number of subjects thus lack a definite COD. Hence, the eventual incorrect diagnoses will pass unnoticed, resources may be allocated based on false presumptions, and diseases, such as dementia and COVID-19, may take longer to discover and cure.

A return to the previous high rates of autopsies is not feasible. However, an autopsy provides an opportunity to investigate the COD, validate clinical diagnoses, detect unexpected aberrations, audit health care, and provide feedback to clinicians to facilitate their continuing education. Thus, the FA/CA is certainly of significance for the health care organisation, and thus, the clinical community should consider their indications for referral to autopsy.

## Strengths and limitations

The main strength of this study is its size, with individual data from over 1.8 million deceased persons over a 20-year period.

The main limitation is that we cannot draw conclusions concerning causality, merely associations.

## Conclusions

The declining autopsy rate in Sweden has been ongoing for decades. A definite COD, i.e. a COD based on autopsy findings, is available for only 12.6% for all deceased during the studied period 1999–2018. We found that men had higher odds compared to females of having an autopsy performed, that people who died from dementia are being autopsied at a very low rate, and that being born outside of Europe was associated with lower odds of undergoing an autopsy.

## Data Availability

The study is based on data from the Swedish cause of death registry. Since it might be possible to identify some individuals from unique combinations of variable values, the data cannot be made publicly available.
